# Molecular mimicry between Zika virus and central nervous system inflammatory demyelinating disorders: the role of NS5 Zika virus epitope and PLP autoantigens

**DOI:** 10.1055/s-0043-1768698

**Published:** 2023-05-09

**Authors:** Laise Carolina França, Fabrícia Lima Fontes-Dantas, Diogo Gomes Garcia, Amanda Dutra de Araújo, João Paulo da Costa Gonçalves, Cláudia Cecília da Silva Rêgo, Elielson Veloso da Silva, Osvaldo José Moreira do Nascimento, Fernanda Cristina Rueda Lopes, Alice Laschuk Herlinger, Renato Santana de Aguiar, Orlando da Costa Ferreira Junior, Fernando Faria Andrade Figueira, Jorge Paes Barreto Marcondes de Souza, Joelma Freire De Mesquita, Soniza Vieira Alves-Leon

**Affiliations:** 1 Universidade Federal do Estado do Rio de Janeiro, Programa de Pós-Graduação em Neurologia, Laboratório de Neurociências Translacional, Rio de Janeiro RJ, Brazil.; 2 Universidade do Estado do Rio de Janeiro, Departamento de Farmacologia e Psicobiologia, Rio de Janeiro RJ, Brazil.; 3 Universidade Federal Fluminense, Hospital Universitário Antônio Pedro, Departamento de Neurologia, Niterói RJ, Brazil.; 4 Universidade Federal Fluminense, Hospital Universitário Antônio Pedro, Departamento de Radiologia, Niterói RJ, Brazil.; 5 Universidade Federal do Rio de Janeiro, Instituto de Biologia, Departamento de Genética, Rio de Janeiro RJ, Brazil.; 6 Hospital São Francisco na Providência de Deus, Departamento de Neurologia, Rio de Janeiro RJ, Brazil.; 7 Universidade Federal do Rio de Janeiro, Hospital Universitário Clementino Fraga Filho, Departamento de Neurocirurgia, Rio de Janeiro RJ, Brazil.; 8 Universidade Federal do Estado do Rio de Janeiro, Departamento de Genética e Biologia Molecular, Grupo de Bioinformática e Biologia Computacional, Rio de Janeiro RJ, Brazil.; 9 Universidade Federal do Rio de Janeiro, Hospital Universitário Clementino Fraga Filho, Centro de Referência e Pesquisa em Esclerose Múltipla e Outras Doenças Desmielinizantes Inflamatórias Idiopáticas do SNC, Rio de Janeiro RJ, Brazil.

**Keywords:** Zika Virus, Demyelinating Diseases, Molecular Mimicry, Viral Nonstructural Proteins, Multiple Sclerosis, Zika Virus, Doenças Desmielinizantes, Mimetismo Molecular, Proteínas não Estruturais Virais, Esclerose Múltipla

## Abstract

**Background** Evidence indicates a strong link between Zika virus (ZikV) and neurological complications. Acute myelitis, optic neuritis, polyneuropathy, and encephalomyelitis that mimic inflammatory idiopathic demyelination disorders (IIDD) after ZikV infection have been reported in Brazil.

**Objective** The present study aims to investigate the possible occurrence of molecular mimicry between ZikV antigens and Multiple Sclerosis (MS) autoantigens, the most frequent IIDD of the central nervous system (CNS).

**Methods** A retrospective cohort study with 305 patients admitted due to suspected arbovirus infection in Rio de Janeiro was performed, all subjects were submitted to neurological examination, and a biological sample was collected for serologic and molecular diagnostic. Bioinformatics tools were used to analyze the peptides shared between ZikV antigens and MS autoantigens.

**Results** Of 305 patients, twenty-six were positive for ZikV and 4 presented IDD patterns found in MS cases. Sequence homology comparisons by bioinformatics approach between NS5 ZikV and PLP MS protein revealed a homology of 5/6 consecutive amino acids (CSSVPV/CSAVPV) with 83% identity, deducing a molecular mimicry. Analysis of the 3D structures revealed a similar conformation with alpha helix presentation.

**Conclusions** Molecular mimicry between NS5 Zika virus antigen and PLP MS autoantigens emerge as a possible mechanism for IDD spectrum in genetically susceptible individuals.

## Resumo

**Antecedentes** Evidências indicam uma forte ligação entre o vírus Zika (ZikV) e complicações neurológicas. Mielite aguda, neurite óptica, polineuropatia e encefalomielite que mimetizam distúrbios inflamatórios de desmielinização idiopáticos (DDII) após infecção por ZikV têm sido relatadas no Brasil.

**Obejtivo** O presente estudo tem como objetivo investigar a possível ocorrência de mimetismo molecular entre antígenos do ZikV e autoantígenos da Esclerose Múltipla (EM), a DDII mais frequente do sistema nervoso central (SNC).

**Métodos** Foi realizado um estudo de coorte retrospectivo com 305 pacientes internados por suspeita de infecção por arbovírus no Rio de Janeiro, todos os indivíduos foram submetidos a exame neurológico e coleta de amostra biológica para diagnóstico sorológico e molecular. Ferramentas de bioinformática foram usadas para analisar os peptídeos compartilhados entre antígenos do ZikV e autoantígenos da EM.

**Resultados** Dos 305 pacientes, vinte e seis foram positivos para ZikV e 4 apresentaram padrão IDD encontrado em casos de EM. As comparações de homologia de sequência por abordagem de bioinformática entre a proteína NS5 ZikV e PLP EM revelaram uma homologia de 5/6 aminoácidos consecutivos (CSSVPV/CSAVPV) com 83% de identidade, deduzindo um mimetismo molecular. A análise das estruturas 3D revelou uma conformação semelhante com apresentação em alfa-hélice.

**Conclusões** O mimetismo molecular entre o antígeno NS5 do vírus Zika e o autoantígeno PLP da EM surge como um possível mecanismo para o espectro IDD em indivíduos geneticamente suscetíveis.

## INTRODUCTION

Over 80% of Zika Virus (ZikV) infections in humans are asymptomatic. Typical symptoms can include rash, fever, joint pain, and conjunctivitis for a period of 7 days. The outbreak of ZikV has increased the occurrence of long term neurological complications, such as Guillain-Barré syndrome, acute flaccid paralysis, and meningoencephalitis. 1 In addition, ZikV was detected by serology in cerebrospinal fluid (CSF), molecular and histopathological analysis of the brain, and amniotic fluid of microcephalic fetuses. 2 3 ZikV has also been associated with central nervous system (CNS) inflammatory demyelinating disorders (IDD) including optic neuritis, 4 neuromyelitis optica spectrum disorders (NMOSD), 5 transverse myelitis and acute disseminated encephalomyelitis (ADEM). 2 Our group has recently published a case in which the coexistence of the virus in the CNS of an MS patient led to the development of an ADEM-like episode. 6

Besides its direct neurotropic effect, 7 it is believed that ZikV may function as a trigger leading to the development of an immune-mediated injury against many parts of the CNS. 8 ZikV has already been related to the development of several autoimmune conditions. 9 In Guillain-Barre syndrome (GBS), for example, the molecular mimicry between glycolipids and surface molecules of the virus has explained the majority of cases. 10 Interestingly, ZikV is commonly associated with magnetic resonance imaging (MRI) lesions distributed in space and time, regarding heterogeneous gadolinium enhancement, as seen in the MS criteria. 11 Moreover, serum positivity for autoantibodies against myelin oligodendrocyte glycoprotein (MOG), a specific antibody against the myelin sheath was recently associated with ZikV. 12 As many radiological and clinical aspects of ZikV infection may mimic IIDD, patients can be misdiagnosed. MS is the most frequent IIDD of the CNS, 13 and several evidences have shown that molecular mimicry is a possible epigenetic mechanism in genetically susceptible individuals. 14

To investigate the mechanisms of ZikV induced neurological manifestations, it is essential to use various reproducible *in vitro* models and bioinformatics tools capable of recapitulating complex neurodevelopmental disorders, in an attempt to find specific targets. The molecular mechanisms underlying these conditions in adults are not clear. Focusing on the MS-like pattern, the present study investigated the possible occurrence of molecular mimicry between ZikV antigens and MS autoantigens. The underlying rationale is that shared peptides between pathogen and human host may lead to a break in immune tolerance through a cross-reactivity phenomenon. 15

## METHODS

### Study population and biological samples

A retrospective cohort study was performed with patients admitted in neurology service of three university hospitals and referred by Laboratório Central Noel Nutels (LACEN) in Rio de Janeiro. This work was approved by the National Council for Ethics in Research (CAAE 69411317.6.0000.5258). All subjects signed an informed consent agreeing to participate in this research. From 2016 to 2019, 305 patients with suspected arbovirus infection were evaluated by a multidisciplinary team. Complete physical and neurological examination was performed and, when necessary, MRI was requested. Biological sample (blood, urine, and CSF) was collected on admission and, according to clinical indication, tested by serology and/or ZikV molecular diagnostic.

### Sequence analysis

Peptide sharing between ZikV antigens and MS autoantigens was analyzed as follows: A viral polyprotein library was constructed using the major viral antigens reported in the literature and protein sequences available in NCBI Protein Reference Sequences ( https://www.ncbi.nlm.nih.gov/protein ). An MS autoantigen library was constructed at random through UniProtKB Database ( www.uniprot.org/ ) using ‘Multiple Sclerosis’ as a keyword. The result was filtered and only the proteins confirmed as autoantigens were collected. ZikV polyproteins and MS autoantigens identified are outlined in Table 1 . The two libraries were analyzed for matches using BLASTP ( https://blast.ncbi.nlm.nih.gov/Blast.cgi ) and sequence alignment was done using EMBOSS ( https://www.ebi.ac.uk/Tools/psa/emboss_water/ ).

**Table 1:** ZikV polyproteins and human MS autoantigens-related proteins

ZikV polyproteins	Multiple sclerosis autoantigens
Chain B, Full-length Ns1 Structure of Zika Virus From 2015 Brazil Strain PDB: 5GS6_B	myelin oligodendrocyte glycoprotein (MOG) UniProtKB- Q16653
Chain A, Full-length Ns1 Structure of Zika Virus From 2015 Brazil Strain PDB: 5GS6_A	myelin basic protein (MBP) UniProtKB- P02686
Chain A, Zika Virus Non-structural Protein 1 (ns1) PDB: 5K6K_A	Myelin associated glycoprotein (MAG) UniProtKB- P20916
Chain B, Zika Virus Non-structural Protein 1 (ns1) PDB: 5K6K_B	Myelin proteolipid protein (PLP) UniProtKB- P60201
Chain A, A Mutation Identified in Neonatal Microcephaly Destabilizes Zika Virus Ns1 Assembly In Vitro PDB: 5 × 8Y_A	
Chain B, A Mutation Identified in Neonatal Microcephaly Destabilizes Zika Virus Ns1 Assembly In Vitro PDB: 5 × 8Y_B	
NS1 [Zika virus] NCBI Reference Sequence: YP_009430301.1	
NS2a NCBI Reference Sequence: YP_009430302.1	
NS2b NCBI Reference Sequence: YP_009430303.1	
NS3 NCBI Reference Sequence: YP_009430304.1	
NS4a NCBI Reference Sequence: YP_009430305.1	
NS4b Chain A, Structure of Zika Virus Ns5 NCBI Reference Sequence: YP_009430307.1	
Chain B, Structure of Zika Virus Ns5 PDB: 5TMH_A	
NS5 protein, partial [Zika virus] GenBank: AJD79051.1, AMP44573.1, AMX81921.1, AMX81921.1	
envelope protein E, partial [Zika virus] GenBank: AOX24134.1	
capsid protein C [Zika virus] NCBI Reference Sequence: YP_009430296.1	

### Antigenic prediction

To confirm whether the NS5 ZikV sequence studied has antigenic properties, VaxiJen version 2.0 ( http://www.ddg-pharmfac.net/vaxijen/VaxiJen/VaxiJen.html ) was used. A threshold antigenic score of 0.5 was defined in order to filter probable non-antigenic sequences. Vaxijen server performs alignment-independent prediction, which is based on auto cross covariance (ACC) transformation of protein sequences into uniform vectors of principal amino acid properties.

### 3D comparative modelling

The 3D models were built using the Swiss-Model, an online modeling server ( https://swissmodel.expasy.org/ ). The template modeling scores (TM-scores) and root mean square deviations (RMSDs) of the NS5 ZikV and PLP MS three-dimensional overlap were calculated using TM-Align.

## RESULTS

### Inflammatory demyelinating disorder phenotypes in patients with ZikV infection

**Figure 1:**
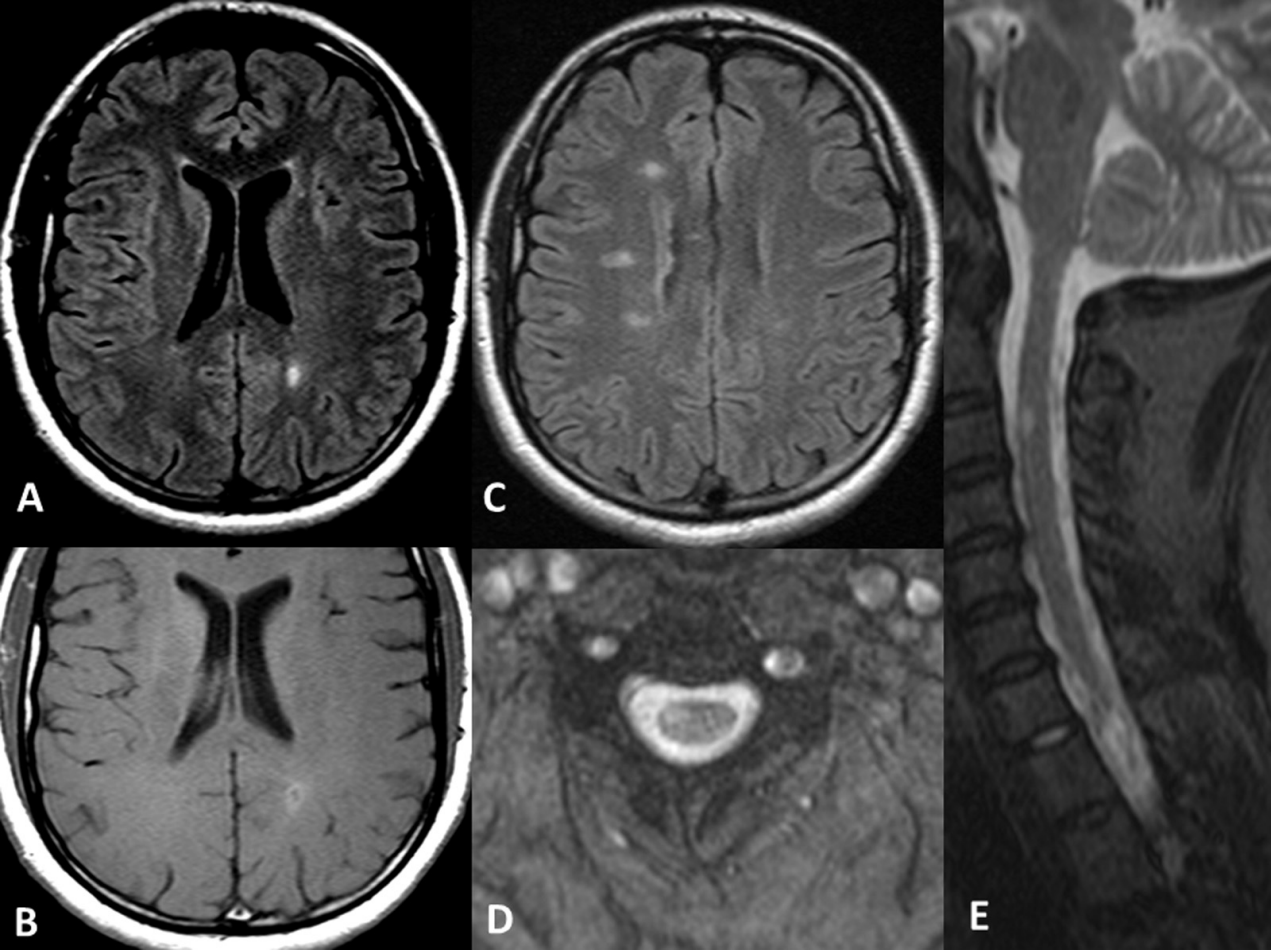
A focal subcortical hyperintense FLAIR lesion ( **A** ) with contrast enhancement ( **B** ) is observed in conjunction with other periventricular and pericalosal bright lesions ( **C** ), similar to Dawson's fingers described for MS disease. Cervical lesions follow the same pattern, eccentrically located in the T2* axial plane ( **D** ) and extending for one vertebral body dimension on the sagittal STIR cervical image ( **E** ).

**Figure 2:**
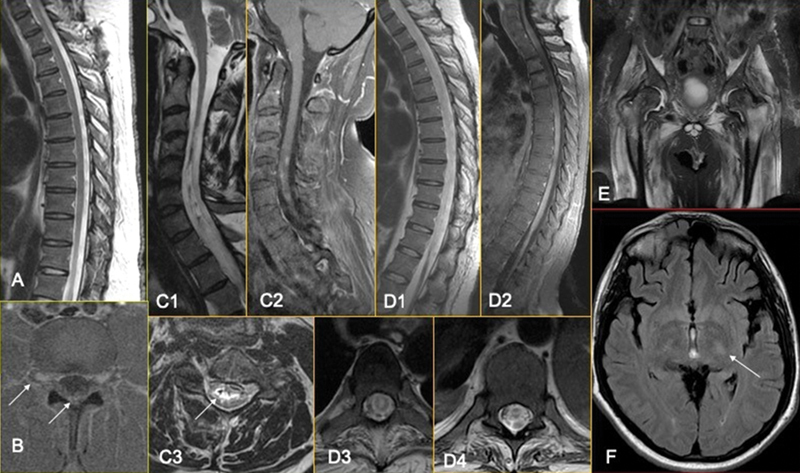
Initial dorsal MRI ( **A** ) was normal, and a significant contrast enhancement was observed in the axial T1 fat-sat image of the lumbar spine, involving the dorsal ganglia and the lumbo-sacral plexus inside the spine canal ( **B** ). After 3 months of evolution, the patient developed longitudinal extensive transverse myelitis ( **C, D** ), already with focal tapering of the cervical/dorsal transition on sagittal STIR (C1), remembering a sequel area. The lesion was centrally located (C3, D3), with anterior horn involvement (D4) and signals of previous bleeding inside the central canal (C3). A patch and irregular contrast enhancement were noticed along the sequel area (C2) and along the entire dorsal spinal cord (D2). Consequent muscle denervation was observed in the coronal STIR of the pelvic girdle muscles ( **E** ) and ascendant cortical-spinal tract degeneration consequent to the spinal cord damage on FLAIR axial images ( **F** ).

**Figure 3:**
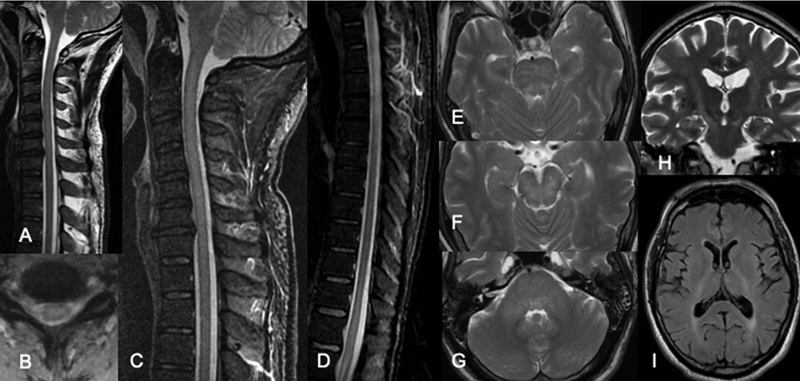
Cervical spinal cord sagittal T2 ( **A** ) shows extensive continuous high signal intensity lesion affecting the hole diameter of the spinal cord on axial T2* images ( **B** ), best identified on sagittal STIR ( **C** ). The extent of more than 3 vertebral bodies was confirmed, as well as the involvement of the medullary cone on sagittal STIR ( **D** ). Brain lesions were mainly detected affecting the brain stem on axial T2 images, including the posterior aspect of the mesencephalon ( **E** ), pons ( **F** ), and the medial cerebellar peduncle ( **G** ). Two years follow up brain images show hypersignal intensity on coronal T2 ( **H** ) and axial FLAIR ( **I** ) images located in the cortical-spinal tract, mostly associated with retrograde degeneration within the spinal cord lesions.

**Figure 4:**
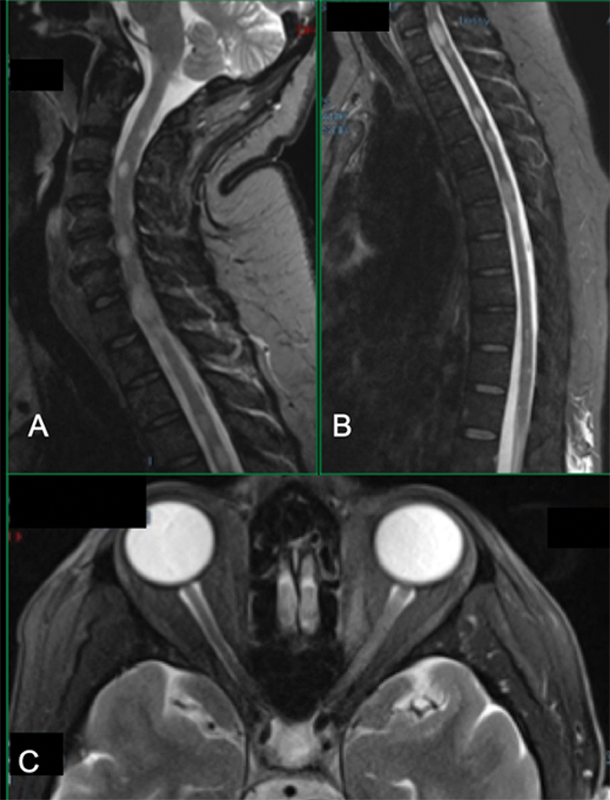
Sagittal STIR cervical ( **A** ) and dorsal ( **B** ) spinal cords have multiple small tumefactive bright lesions, randomly affecting all main cords tracts, diffusively distributed. Axial T2-fat suppressed at the orbital area shows bright thickening of the intra-orbital extent of the left optic nerve, reflecting extensive optic neuritis.

A total of 305 patients were evaluated. 26 were positive for ZikV and the remaining were diagnosed with either Dengue or Chikungunya. Out of the ZikV positive patients, 4 were classified as having IDD of the CNS requiring differential diagnosis with MS. Clinical examination, imaging, electrophysiologic, and laboratory findings of these patients are exposed in Table 2 and Figures 1 - 4 .

**Table 2:** Clinical and laboratory findings in patients with Zika virus–associated Multiple Sclerosis-like manifestations

Clinical presentation	Patient 1	Patient 2	Patient 3	Patient 4
**Age**	30	51	48	57
**Sex**	Male	Male	Male	Female
**Medical history**	EM	None	None	None
**Viral prodrome**	Fever and myalgia	Acute fever and rash	Acute fever and rash	Fever, intense myalgia, and skin rash
**Neurologic symptoms**	Acute encephalomyelitis with drowsiness, mental confusion, locomotor disorders and diplopia. ADEM	Paraparesis, that evolved into tetraparesis. ADEM	Agitation and disorientation preceded, acute encephalomyelitis	Visual loss and walking impairment
**Time from viral prodrome to neurologic symptoms**	5 days	11 days	10 days	11 days
**Neurologic examination**	Hypoesthesia right upper limb and papillitis,	Dysarthria, tetraparesis and drowsiness	Tetraparesis, disorientation and decreased consciousness level	Spastic symmetric crural paraplegia, papilemema, left visual loss
**Diagnostic studies**				
**ZikV RT-PCR**	Negative	Negative	Negative	Positive
**Igm ZikV**	Positive in the serum and urine	Positive in the serum	Positive in the serum	Positive in the serum
**Igg ZikV**	Positive in the serum, urine and LCR	Positive in the serum	Positive in the serum	Negative in the serum
**CSF**	8 leukocyte/mm ^3^ , 27 mg/dl protein, OCB positive, IgG index 0,88	0 leukocytes/mm ^3^ , 27 mg/dl protein.	15 leukocytes/mm ^3^ , 71 mg/dL protein, and 55 mg/dL glucose	9 leukocyte/mm ^3^ , 64 mg/dl protein, OCB negative, AQP4 Ab Negative

Patient 1 presented with headache, optical neuritis, and cervical myelitis associated with a cervical lesion ( Figure 1 E ) and asymptomatic multifocal brain lesions on MRI, one of which had gadolinium enhancement. This distribution of brain lesions, paired with positive oligoclonal bands (OCB) found on CSF analysis, resembles the pattern usually found in MS ( Figure 1 A-D and Table 1 ).

Patient 2 had a diagnosis of acute flaccid paraplegia 11 days after a viral prodrome, and 3 months later developed tetraparesis associated with longitudinal extensive transverse myelitis ( Figure 2C and 2D ), centrally located ( Figure 2 C3, D3 ), with focal tapering of the cervical/dorsal transition on sagittal STIR ( Figure 2 C1 ), resembling the extension and sequelae areas usually seen in NMOSD. Furthermore, the lesion had anterior horn involvement ( Figure 2 D4 ).

Patient 3 presented with tetra paresis and ataxia associated with brain lesions mainly affecting the brainstem on axial T2 images, including the posterior aspect of the mesencephalon ( Figure 3E ), pons ( Figure 3F ) and the medial cerebellar peduncle ( Figure 3G ).

Patient 4 presented with optic neuritis and multifocal myelitis with cervical and dorsal lesions, as usually found in a first manifestation of MS ( Figures 4A and 4B ).

### Sequence sharing between ZikV polyproteins and MS autoantigens

The bioinformatics approach identified an 83% identity between the NS5 antigen of ZikV and PLP MS autoantigen, deducing the molecular mimicry among them. Although statistically non-significant, it was also possible to observe a 67% identity between NS3 antigen of ZikV and MOG MS autoantigen. The identity results between all sequences are depicted in Table 3 . In addition, sequence analysis of NS5 using VaxiJen version 2.0 resulted in a score of 0.5091, confirming the antigenicity of the sequence studied.

**Table 3:** Results of alignment between ZikV polyproteins and MS autoantigens

Autoantigens	Polyproteins	Identity
MOG	NS1	50%
MOG	Chain B, Full-length Ns1 Structure of Zika Virus From 2015 Brazil Strain	43%
MOG	Chain A, Full-length Ns1 Structure of Zika Virus From 2015 Brazil Strain	43%
MOG	Chain A, Zika Virus Non-structural Protein 1 (ns1)	43%
MOG	Chain B, Zika Virus Non-structural Protein 1 (ns1)	43%
MOG	Chain A, A Mutation Identified in Neonatal Microcephaly Destabilizes Zika Virus Ns1 Assembly In Vitro	36%
MOG	Chain B, A Mutation Identified in Neonatal Microcephaly Destabilizes Zika Virus Ns1 Assembly In Vitro	36%
MOG	NS2a	0%
MOG	NS2b	0%
MOG	NS3	67%
MOG	NS4a	50%
MOG	NS4b	29%
MOG	Chain A, Structure of Zika Virus Ns5	50%
MOG	Chain B, Structure of Zika Virus Ns5	50%
MOG	NS5 protein, partial [Zika virus]	50%
MOG	envelope protein E, partial	50%
MOG	capsid protein C	35%
PBM	Chain B, Full-length Ns1 Structure of Zika Virus From 2015 Brazil Strain	0%
PBM	Chain A, Full-length Ns1 Structure of Zika Virus From 2015 Brazil Strain	0%
PBM	Chain A, Zika Virus Non-structural Protein 1 (ns1)	0%
PBM	Chain B, Zika Virus Non-structural Protein 1 (ns1)	0%
PBM	Chain A, A Mutation Identified in Neonatal Microcephaly Destabilizes Zika Virus Ns1 Assembly In Vitro	0%
PBM	Chain B, A Mutation Identified in Neonatal Microcephaly Destabilizes Zika Virus Ns1 Assembly In Vitro	0%
PBM	NS1	0%
PBM	NS2a	50%
PBM	NS2b	21%
PBM	NS3	33%
PBM	NS4a	33%
PBM	NS4b	40%
PBM	Chain A, Structure of Zika Virus Ns5	32%
PBM	Chain B, Structure of Zika Virus Ns5	32%
PBM	NS5 protein, partial	30%
PBM	envelope protein E, partia	40%
PBM	capsid protein C	0%
MAG	Chain B, Full-length Ns1 Structure of Zika Virus From 2015 Brazil Strain	0%
MAG	Chain A, Full-length Ns1 Structure of Zika Virus From 2015 Brazil Strain	0%
MAG	Chain A, Zika Virus Non-structural Protein 1 (ns1)	0%
MAG	Chain B, Zika Virus Non-structural Protein 1 (ns1)	0%
MAG	Chain A, A Mutation Identified in Neonatal Microcephaly Destabilizes Zika Virus Ns1 Assembly In Vitro	0%
MAG	Chain B, A Mutation Identified in Neonatal Microcephaly Destabilizes Zika Virus Ns1 Assembly In Vitro	0%
MAG	NS1	0%
MAG	NS2a	50%
MAG	NS2b	21%
MAG	NS3	33%
MAG	NS4a	33%
MAG	NS4b	40%
MAG	Chain A, Structure of Zika Virus Ns5	32%
MAG	Chain B, Structure of Zika Virus Ns5	32%
MAG	NS5 protein, partial	30%
MAG	envelope protein E, partial	40%
MAG	capsid protein C	0%
PLP	Chain B, Full-length Ns1 Structure of Zika Virus From 2015 Brazil Strain	56%
PLP	Chain A, Full-length Ns1 Structure of Zika Virus From 2015 Brazil Strain	56%
PLP	Chain A, Zika Virus Non-structural Protein 1 (ns1)	56%
PLP	Chain B, Zika Virus Non-structural Protein 1 (ns1)	56%
PLP	Chain A, A Mutation Identified in Neonatal Microcephaly Destabilizes Zika Virus Ns1 Assembly In Vitro	0%
PLP	Chain B, A Mutation Identified in Neonatal Microcephaly Destabilizes Zika Virus Ns1 Assembly In Vitro	0%
PLP	NS1	56%
PLP	NS2a	24%
PLP	NS2b	38%
PLP	NS3	46%
PLP	NS4a	0%
PLP	NS4b	50%
PLP	Chain A, Structure of Zika Virus Ns5	30%
PLP	Chain B, Structure of Zika Virus Ns5	30%
PLP	NS5 protein, partial	83%
PLP	envelope protein E, partial	42%
PLP	capsid protein C	20%

### Structural conformation between NS5 ZikV and PLP MS

**Figure 5:**
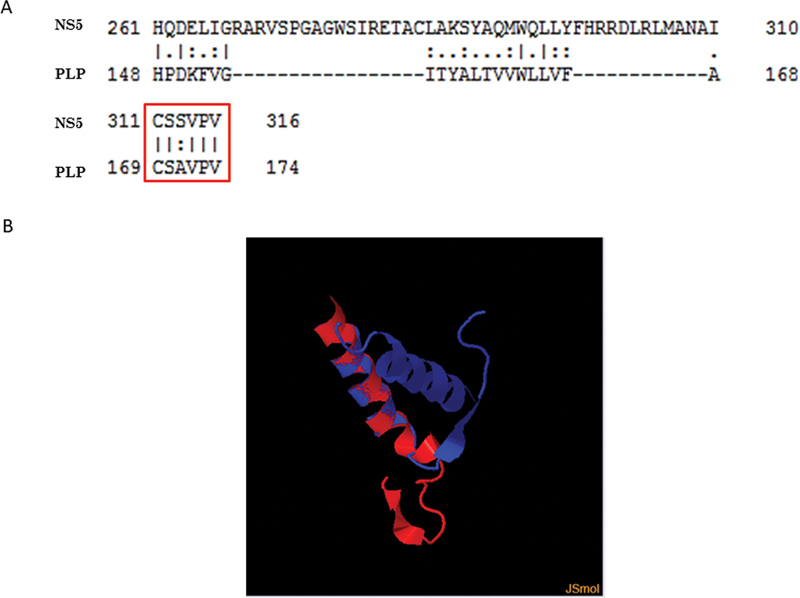
Results of alignment between NS5 antigen of ZikV and PLP MS autoantigens. Alignment of amino acid using EMBOSS needle. Note the motif highlighted has 83% identity (A). Structural alignment between PLP ^131-198^ and NS5 ^281-325^ proteins. In red the PLP ^131-198^ is represented and in blue the NS5 ^281-325^ (B).

In order to predict the 3D structures conformation of the two proteins, TM-Align was used to align them. As Blast P showed us a high identity between a particular region of PLP and NS5, a structural conformation was performed only with that region where the corresponding high identity was obtained PLP ^131-198^ and NS5 ^281-325^ ( Figure 5B ). The CSAVPV sequence which is 83% identity by BlastP, obtained a TM-score of 0.47071 and RMDS of 2.39 and is in the alpha helix structure of both proteins.

## DISCUSSION

Several studies have shown that IDD in CNS can be triggered by viral infection or immunizations. After a variable period of incubation, myelin destruction undergoes courses of remission and exacerbation. MS is a most common disease that compromises CNS myelin sheath. 16

Viral infection can trigger autoimmune diseases through different mechanisms: molecular mimicry, epitope spreading, bystander activation, superantigen production, and inadequate activation of an immune response. 17 Molecular mimicry can be defined as similar structures shared by a host epitope and microorganism or environmental proteins. 17 Using bioinformatics tools, common sequences and structural homology between Chikungunya virus (ChikV) E1 glycoprotein and human HLA-B27 molecule were identified. In addition, the peptides derived from ChikV glycoprotein E1 induced significant inflammation in C57BL/6J mice. 18 Based on proteomic studies and sequence analysis, some evidence has also shown that Dengue Hemorrhagic Fever may be caused by molecular mimicry between different coagulation molecules with prM, E, and NS1 viral proteins. 19 Furthermore, it is already widely proposed that cross-recognition of common viral peptides with myelin antigens induces a molecular mimicry involved in MS development, especially in genetically susceptible individuals. 14

Zika Virus has 7 non structural proteins (NS1, NS2a, NS2b, NS3, NS4a, NS4b, and NS5). Each of them has a paper on the antagonism to the innate immunity. However, the NS5 protein stands out here, as it is the most conserved protein in the flavivirus proteome and it can modulate the host immune response during ZikV infection. 20 NS5 is an antagonist of the interferon response in the host human system and promotes the degradation of STAT2 in ZikV. This same mechanism is well reported in DenV. The importance of NS5 in host immune response modulation and viral replication makes it an attractive target for developing broadly acting antiviral inhibitors. 21

Zika fever is a self-limited disease, still, less than 5% of symptomatic patients may develop neurological manifestations. 22 23

Although patient 1 developed neurological manifestations five days after the first symptoms of viral infection, it was only possible to make the ZikV diagnosis after sixty days, thus explaining the IgM negativity in serum.

A recent case report identified concurrent GBS and ADEM in a 24-year-old woman who developed acute ZikV infection. The authors postulate this case was para-infectious, induced by neurotropism and activation of an immune response against ZikV. 24 This same mechanism is probably involved in the development of this NMOSD phenotype in our patient 2.

Patient 3 could be classified as having a clinical isolated syndrome (CIS) with a high risk of conversion to MS due to the distribution and number of T2 white matter lesions. Although the optical neuritis pattern resembles the one of NMOSD, the spinal cord lesions are MS-like.

Lucchese et al *.* 2016, observed that ZikV antigens are commonly involved in microcephaly and GBS. 129 immunopositive epitopes are reported as having peptide overlap with human proteins that may relate to demyelination and axonal neuropathies. This indicates that cross-reactivity with human proteins might contribute to the mechanisms linking ZikV infection to GBS. 10 The IDD phenotype attributed to ZikV infection seems to mimic MS manifestations. Molecular mimicry is assessed in this study by investigating homologous regions between ZikV antigens and human MS autoantigens using bioinformatics tools. Sequence homology comparisons between NS5 ZikV and PLP MS protein revealed a homology of 5/6 consecutive amino acids CSSVPV/CSAVPV ( Figure 5A ). A study that performed antigenic B-cell epitopes prediction found an antigenic peptide from position 528 to 539 (NAI **CSSVPV** DWV) of ZikV NS5, which had the maximum residual score of 1.203 and might present a preliminary set of peptides for future vaccine development against ZikV. 25 Calculating the TM-score of NS5 ZikV and PLP MS 3D structures demonstrated that both proteins are in almost the same fold, both are in alpha helix and they have topological similarity ( Figure 5B ). 26

Interestingly, ZikV African (MR766) lineage strain, revealed exactly the same Human PLP sequence (CSAVPV), and recombinant NS5 proteins from Africa and from Brazil revealed similar levels of RNA synthesis. 27 It is already known that the MR766 strain is more virulent and causes more severe brain damage than the current Asian lineage and dengue virus. 28 When inoculated subcutaneously in adult transgenic mice (knockout) C57BL/6 Stat2 ^-/-^ MR766 strain induces short episodes of severe neurological symptoms, followed by lethality. Furthermore, this strain was able to induce higher levels of inflammatory cytokines and markers associated with cellular infiltration into the brain of infected mice. 29 Li et al. 2019, observed that MR766 strain and epidemic Brazilian (BR15 and ICD) ZikV strains are different in viral attachment to host neuronal cells, viral permissiveness, and replication, as well as in the induction of cytopathic effects. 30

Autoreactivity to PLP in patients with MS has been investigated in human and animal model by various groups worldwide. 31 A recent study involving PLP's Epitopes involved in MS, found CSAVPV (in PLP ^161-177^ residues) among the most immunogenic regions of PLP. 32 In addition, the crystal structure of the NS5 ZikV protein reveals a conserved domain conformation of Flaviviruses, a genus that includes a variety of human pathogens such as dengue virus, yellow fever virus, WNV, Spondweni virus, and the Japanese encephalitis virus. 33 So, the presence of high identity between NS5 ZikV and PLP, an autoantigen widely implicated in the pathogenesis of MS, 34 leads us to postulate that molecular mimicry may have a role in the development of inflammatory demyelinating damage, a hallmark of the IDD produced by this genus of virus.

Both genetic and environmental factors have been shown to contribute to the pathogenesis of autoimmune diseases. It is well-established that the HLA-DR15 haplotype bears the strongest association with MS. 35 In a Brazilian study, it was observed that the presence of HLA-DRB1*1501 allele confers an ethnicity-dependent MS susceptibility in Caucasian patients and that the HLA-DQB1*0602 allele confers an ethnicity independent susceptibility. 36 Using HLA class II transgenic (Tg) mice, several studies have demonstrated HLA-DR-dependent disease following immunization by MBP, PLP, or MOG. 37 38 However, it was observed that HLA-DRB1*1501 Tg mice were refractory to disease induction by overlapping PLP peptides, while HLA-DQB1*0602 Tg mice were susceptible to disease induction by PLP ^139-151^ and PLP ^175-194^ peptides. 39 It has been seen that Both PLP ^139-151^ and PLP ^178-191^ epitopes are key targets of T-cells, and are increased in MS patients versus healthy controls. 40 However, this does not mean that PLP ^161-177^ residues are not encephalitogenic-related, but that they need further animal and human model studies. Therefore, PLP autoimmunity and HLA haplotype have been strongly associated with lesion localization, as well as remission and relapse rates in MS. 41

In conclusion, the concept of molecular mimicry remains a viable hypothesis for understanding the genetics, epigenetics, and environmental involvement in the pathogenic mechanisms of IDD. Studies using bioinformatics tools further encourage the identification of molecules that could be used in the development of either diagnostic or prognostic biomarkers. We found that NS5 ZikV presented a high identity with PLP MS autoantigen, and both are structurally similar to alpha helix chains. These findings may justify IDD CNS manifestations following ZikV infection, as in the 4 cases here reported. Further investigation is required to understand whether PLP ^161-177^ residues are encephalitogenic and how the recognition of NS5 epitopes by HLA molecules drives the pathogenic T-cell autoimmune response *in vivo.*

## References

[1] Baud D Gubler D J Schaub B Lanteri M C Musso D An update on Zika virus infectionLancet 2017;390(10107):2099–2109. Doi: 10.1016/S0140-6736(17)31450-2 [Internet] 10.1016/S0140-6736(17)31450-2 28647173

[2] Galliez R M Spitz M Rafful P P Zika virus causing encephalomyelitis associated with immunoactivation Open Forum Infect Dis 2016 3 04 ofw203 10.1093/ofid/ofw203 28053996 PMC5193179

[3] Mlakar J Korva M Tul N Zika Virus Associated with Microcephaly N Engl J Med 2016 374 10 951 958 10.1056/NEJMoa1600651[Internet] 26862926

[4] Singh S Kumar A Ocular Manifestations of Emerging Flaviviruses and the Blood-Retinal Barrier Viruses 2018 10 10 1 20 10.3390/v10100530 PMC6213219 30274199

[5] Aspahan M C Leonhard S E Gomez R S Rocha E da S Vilela M da S Alvarenga P PM https://doi.org/10.1212/CPJ.0000000000000546 10.1212/CPJ.0000000000000546 PMC6382372 30859012

[6] Alves-Leon S V Lima M DR Nunes P CG Zika virus found in brain tissue of a multiple sclerosis patient undergoing an acute disseminated encephalomyelitis-like episode Mult Scler 2019 25 03 427 430 10.1177/1352458518781992 30226115

[7] Platt D J Smith A M Arora N Diamond M S Coyne C B Miner J J Zika virus-related neurotropic flaviviruses infect human placental explants and cause fetal demise in mice Sci Transl Med 2018 10 426 eaao7090 10.1126/scitranslmed.aao7090[Internet] 29386359 PMC6136894

[8] Wen J Shresta S T Cell Immunity to Zika and Dengue Viral Infections J Interferon Cytokine Res 2017 37 11 475 479 10.1089/jir.2017.0106 29135369 PMC5695673

[9] Monsalve D M Pacheco Y Acosta-ampudia Y Rodríguez Y Ramírez-santana CZika virus and autoimmunity. One-step forward. Autoimmun Rev [Internet]. 2017;17(S1568-9972):30258–6 Available from:https://doi.org/10.1016/j.autrev.2017.10.008 10.1016/j.autrev.2017.10.008 29037898

[10] Lucchese G Kanduc D Zika virus and autoimmunity: From microcephaly to Guillain-Barré syndrome, and beyond Autoimmun Rev 2016 15 08 801 808 10.1016/j.autrev.2016.03.020[Internet] 27019049

[11] Zare Mehrjardi M Keshavarz E Poretti A Hazin A N Neuroimaging findings of Zika virus infection: a review article Jpn J Radiol 2016 34 12 765 770 10.1007/s11604-016-0588-5 27714487

[12] Neri V C Xavier M F Barros P O Melo Bento C Marignier R Papais Alvarenga R Case Report: Acute Transverse Myelitis after Zika Virus Infection Am J Trop Med Hyg 2018 99 06 1419 1421 10.4269/ajtmh.17-0938 30277201 PMC6283478

[13] Thompson A J Baranzini S E Geurts J Hemmer B Ciccarelli O Multiple sclerosisLancet 2018;391(10130):1622–1636. Doi: 10.1016/S0140-6736(18)30481-1 [Internet] 10.1016/S0140-6736(18)30481-1 29576504

[14] Huynh J L Casaccia P Epigenetic mechanisms in multiple sclerosis: implications for pathogenesis and treatment Lancet Neurol 2013 12 02 195 206 10.1016/S1474-4422(12)70309-5 23332363 PMC3690378

[15] Robinson A P Harp C T Noronha A Miller S D The experimental autoimmune encephalomyelitis (EAE) model of MS: utility for understanding disease pathophysiology and treatment Handb Clin Neurol 2014 122 173 189 10.1016/B978-0-444-52001-2.00008-X 24507518 PMC3981554

[16] Waubant E Ponsonby A L Pugliatti M Hanwell H Mowry E M Hintzen R Q Environmental and genetic factors in pediatric inflammatory demyelinating diseases Neurology 2016 87(9, Suppl 2)S20 S27 10.1212/WNL.0000000000003029 27572857

[17] Oldstone M B Molecular mimicry and immune-mediated diseases FASEB J 1998 12 13 1255 1265 10.1096/fasebj.12.13.1255 9761770 PMC7164021

[18] Reddy V Desai A Krishna S S Vasanthapuram R Molecular Mimicry between Chikungunya Virus and Host Components: A Possible Mechanism for the Arthritic Manifestations PLoS Negl Trop Dis 2017 11 01 e0005238 10.1371/journal.pntd.0005238 28125580 PMC5268390

[19] Lin Y-S Yeh T-M Lin C-F Molecular mimicry between virus and host and its implications for dengue disease pathogenesis Exp Biol Med (Maywood) 2011 236 05 515 523 10.1258/ebm.2011.010339 21502191

[20] NG https://doi.org/10.1021/acsinfecdis.8b00373

[21] Shi Y Gao G F Structural Biology of the Zika Virus Trends Biochem Sci 2017 42 06 443 456 28318966 10.1016/j.tibs.2017.02.009

[22] Meltzer E Leshem E Lustig Y Gottesman G Schwartz E The Clinical Spectrum of Zika Virus in Returning Travelers Am J Med 2016 129 10 1126 1130 10.1016/j.amjmed.2016.04.034[Internet] 27260832

[23] Beckham J D Pastula D M Massey A Tyler K L Zika virus as an emerging global pathogen: Neurological complications of zika virus JAMA Neurol 2016 73 07 875 879 10.1001/jamaneurol.2016.0800 27183312 PMC5087605

[24] Román G C Anaya J-M Mancera-Páez Ó Pardo-Turriago R Rodríguez Y Concurrent Guillain-Barré syndrome, transverse myelitis and encephalitis post-Zika: A case report and review of the pathogenic role of multiple arboviral immunity J Neurol Sci 2019 396 84 85 10.1016/j.jns.2018.10.032[Internet] 30423542

[25] Mirza M U Rafique S Ali A Towards peptide vaccines against Zika virus: Immunoinformatics combined with molecular dynamics simulations to predict antigenic epitopes of Zika viral proteins Sci Rep 2016 6(July):37313 10.1038/srep37313 27934901 PMC5146661

[26] Zhang Y Skolnick J TM-align: a protein structure alignment algorithm based on the TM-score Nucleic Acids Res 2005 33 07 2302 2309 10.1093/nar/gki524 15849316 PMC1084323

[27] Zhao B Yi G Du F Structure and function of the Zika virus full-length NS5 protein Nat Commun 2017 8 14762 10.1038/ncomms14762[Internet] 28345656 PMC5378950

[28] Shao Q Herrlinger S Zhu Y-N Yang M Goodfellow F Stice S L https://doi.org/10.1242/dev.156752 10.1242/dev.156752 PMC5719247 28993398

[29] Tripathi S Balasubramaniam V RMT Brown J A Mena I Grant A https://doi.org/10.1371/journal.ppat.1006258

[30] EZV https://doi.org/10.3390/v11020157

[31] Greer J M Autoimmune T-cell reactivity to myelin proteolipids and glycolipids in multiple sclerosis Mult Scler Int 2013 2013 151427 10.1155/2013/151427 24312732 PMC3839122

[32] Zamanzadeh Z Ataei M Nabavi S M Ahangari G Sadeghi MIn Silico Perspectives on the Prediction of the PLP ' s Epitopes involved in Multiple Sclerosis. Natl Inst Genet Eng Biotechnol [Internet]. 2017;15(1):10–21. Available from:http://dx.doi.org/10.15171/ijb.1356 10.15171/ijb.1356 PMC5582249 28959348

[33] Wang B Tan X F Thurmond S The structure of Zika virus NS5 reveals a conserved domain conformation Nat Commun 2017 8 14763 10.1038/ncomms14763[Internet] 28345600 PMC5378951

[34] Kuhlmann T Ludwin S Prat A Antel J Brück W Lassmann H An updated histological classification system for multiple sclerosis lesions Acta Neuropathol 2017 133 01 13 24 27988845 10.1007/s00401-016-1653-y

[35] Svejgaard A The immunogenetics of multiple sclerosis Immunogenetics 2008 60 06 275 286 10.1007/s00251-008-0295-1 18461312

[36] Alves-Leon S V Papais-Alvarenga R Magalhães M Alvarenga M Thuler L CS Fernández y Fernandez O Ethnicity-dependent association of HLA DRB1-DQA1-DQB1 alleles in Brazilian multiple sclerosis patients Acta Neurol Scand 2007 115 05 306 311 10.1111/j.1600-0404.2006.00750.x 17489940

[37] Mangalam A K Khare M Krco C Rodriguez M David C Identification of T cell epitopes on human proteolipid protein and induction of experimental autoimmune encephalomyelitis in HLA class II-transgenic mice Eur J Immunol 2004 34 01 280 290 10.1002/eji.200324597 14971054

[38] Rich C Link J M Zamora A Myelin oligodendrocyte glycoprotein-35-55 peptide induces severe chronic experimental autoimmune encephalomyelitis in HLA-DR2-transgenic mice Eur J Immunol 2004 34 05 1251 1261 10.1002/eji.200324354 15114658

[39] Kaushansky N Altmann D M David C S Lassmann H Ben-Nun A DQB1*0602 rather than DRB1*1501 confers susceptibility to multiple sclerosis-like disease induced by proteolipid protein (PLP) J Neuroinflammation 2012 9 01 29 10.1186/1742-2094-9-29 22316121 PMC3344688

[40] Bielekova B Sung M-H Kadom N Simon R McFarland H Martin R Expansion and functional relevance of high-avidity myelin-specific CD4+ T cells in multiple sclerosis J Immunol 2004 172 06 3893 3904 10.4049/jimmunol.172.6.3893 15004197

[41] Greer J M Csurhes P A Muller D M Pender M P Correlation of blood T cell and antibody reactivity to myelin proteins with HLA type and lesion localization in multiple sclerosis J Immunol 2008 180 09 6402 6410 18424764 10.4049/jimmunol.180.9.6402

